# Leaf morphology and chlorophyll fluorescence characteristics of mulberry seedlings under waterlogging stress

**DOI:** 10.1038/s41598-021-92782-z

**Published:** 2021-06-28

**Authors:** Liangyi Rao, Siyuan Li, Xue Cui

**Affiliations:** 1 College of Soil and Water Conservation, Beijing Forestry University, Beijing, 100083 China; 2 Engineering Research Center of Forestry Ecological Engineering of Ministry of Education, Beijing, 100083 China

**Keywords:** Ecology, Plant sciences

## Abstract

Because of its high flooding tolerance, in recent years, mulberry has become a tree species that is used in plant restoration in impact zones in reservoir areas. Therefore, 3-year-old potted forage mulberry seedlings were used to investigate the mechanism of mulberry adaptation to flooding stress. An indoor simulated flooding method was adopted to study the morphology of mulberry seedling leaves and the changes in leaf chlorophyll fluorescence parameters and fluorescence imaging under different flooding times and depths. The results showed that the leaves of mulberry seedlings treated with shallow submergence remained healthy during the flooding period, while the leaves of mulberry seedlings treated with half submergence and full submergence showed different degrees of waterlogging symptoms in the middle and late flooding periods and formed adventitious roots at the base of the stem. Most of the chlorophyll fluorescence parameters decreased at the beginning of flooding, but the steady-state degree of closure of PS II reaction centres (1-qP_Lss) increased significantly. In the later stage of flooding, the fluorescence parameters showed relatively stable trends. Based on these results, we conclude that mulberry has high flooding tolerance due to a combination of morphological and physiological responses.

## Introduction

Mulberry (*Morus alba* L.) is a deciduous tree or shrub in the mulberry family, that has a well-developed root system and has been widely cultivated worldwide for a long time^1^. Several studies have reported that mulberry trees have good adaptability to waterlogging stress, and those planted in the water-level-fluctuating zone (WLFZ) of the Three Gorges Reservoir area (TGRA) at elevations ranging from 170 to 175 m survived and grew well after several winter floods^2^. The germination rate of mulberry seedlings at an elevation above 170 m in the WLFZ reached 62.6% after 90 days of submerged flooding. Although mulberry seedlings at elevations below 165 m experienced very long periods of flooding (214 days), most mulberry seedlings from that area could still germinate normally due to their developed root systems^3^.

Other studies have also pointed out that mulberry trees exhibit high drought tolerance by increasing their root absorptive area, reducing stomatal conductance and enhancing their capacity for water retention^4^. In addition, it was found that mulberry trees could slow surface runoff, protect soil resources and isolate the heavy metal pollutants in the TGRA^5^. Therefore, mulberry trees are considered to be candidate species for vegetation restoration in the WLFZ of the TGRA.

Photosynthesis in plants is highly sensitive to biotic and abiotic stresses^6^, so studying the photosynthetic characteristics of plants is an effective way to reveal the mechanisms of their adaptation to different environments^7^. The dynamic changes in chlorophyll fluorescence in plants can characterize the local changes in the function of the photosynthetic apparatus^8^. Chlorophyll fluorescence imaging technology has been recognized as a non-destructive and non-invasive technique for detecting the relationship between photosynthesis and the plant environment^9,10^. In recent years, much research has been carried out on the effects of adversity stress on plant fluorescence characteristics. For example, the effects of waterlogging stress on the photosynthetic characteristics of *Pterocarya stenoptera* C. DC., *Salix integra* CV. ‘Hakuro nishki’, *Hemarthria altissima* (Poir.) Stapf et C. E. Hubb., *Phragmites australis* (Cav.) Trin. ex Steud. and *Distylium chinense* (Fr.) Diels^11–15^; the effects of salt stress on the photosynthetic characteristics of *Morus alba* L., *Populus tomentosa* Carr., *Celtis sinensis* Pers. and *Ulmus pumila* L. seedlings^16–18^, and the effects of drought stress on the photosynthetic characteristics of rootstocks of *Vitis vinifera* Linn. and leaves of *Fragaria* × *ananassa* Duch.^19,20^ have been studied. However, studies on the chlorophyll fluorescence characteristics of mulberry seedlings under waterlogging stress have not been reported. The main objectives of this study were to quantify the leaf morphology and chlorophyll fluorescence characteristics of 3-year-old mulberry seedlings under different depths of waterlogging stress and to reveal their photosynthetic physiological and ecological responses under different levels of waterlogging stress.

## Results

### Effects of waterlogging stress on leaf morphology in mulberry seedlings

**Figure 1:**
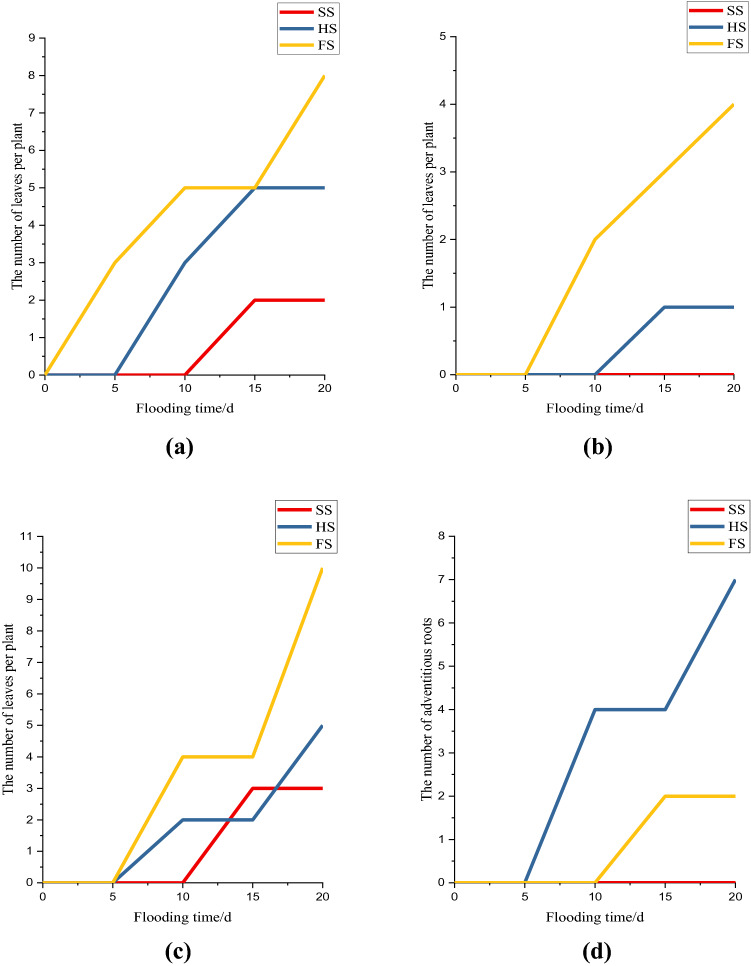
Effect of submergence stress on leaf morphology in *Morus alba*: (**a**) The number of curled or wilted leaves per plant; (**b**) The number of brown spots or rotten leaves per plant; (**c**) The number of fallen leaves per plant; (**d**) The number of adventitious roots. This figure was drawn using Origin Pro 2021 v. 9.8.0.200.

Figure 1 shows the change in the leaf morphology of mulberry seedlings under different submergence depths. The results showed that the seedlings under both SS and HS could grow well, and there were 3 slightly wilted leaves on average under FS. There were 3 wilted leaves and 2 defoliated leaves on average in the HS group after 10 days of flooding, and a few adventitious roots began to appear at the base of the stem. In the SS group, there slight wilting and falling of mulberry leaves were observed on the 15th day after submergence, and there were 5 wilting leaves and a few adventitious roots per plant. In the SS group, there were 3 defoliated leaves and 2 wilted leaves per mulberry seedling, and no adventitious roots developed. The HS group showed an average of 7 adventitious roots per plant. Additionally, there were 8 wilted leaves, 10 defoliated leaves and 4 brown spots per plant under HS.

### Effects of waterlogging stress on initial fluorescence (Fo), and maximum fluorescence (Fm) under dark adaptation in mulberry leaves

**Figure 2:**
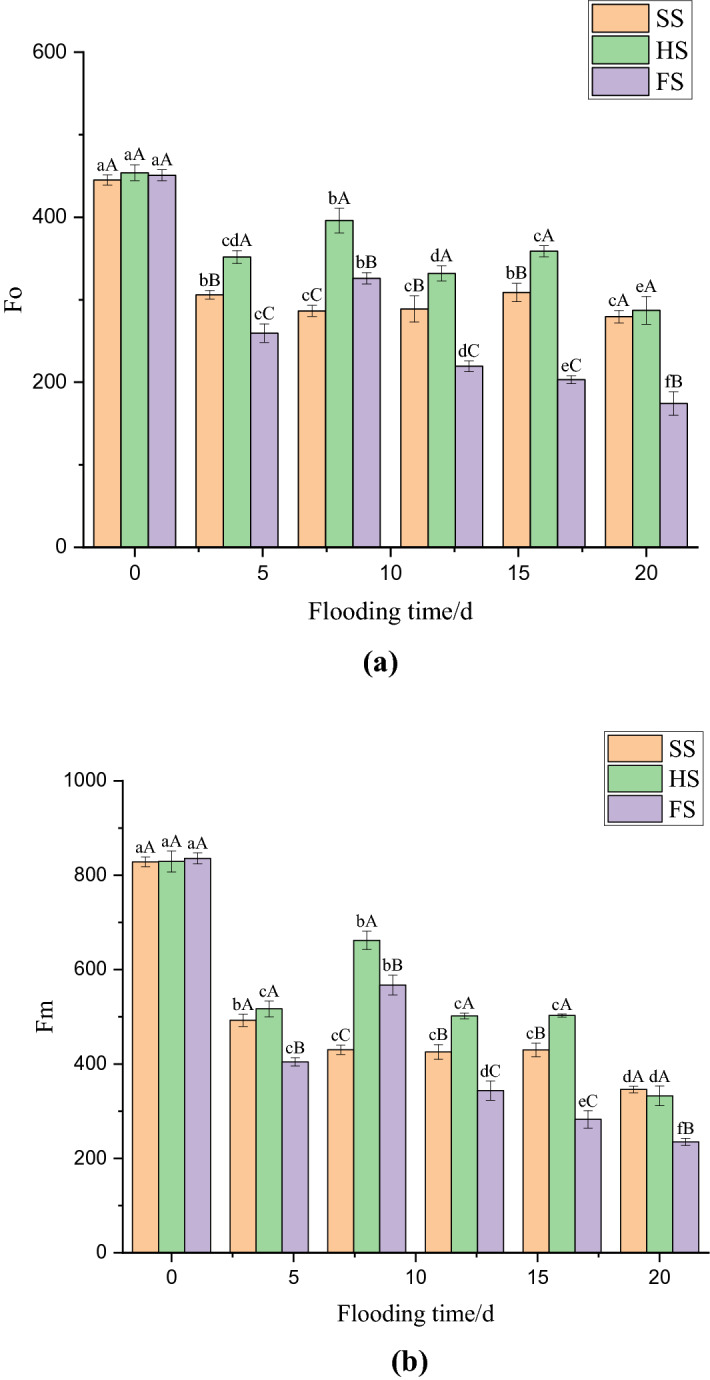
Effect of submergence stress on Fo and Fm in *Morus alba.* Different uppercase letters indicate that the means of the different groups at the same time are significantly different (*p* < 0.05), and different lowercase letters indicate that the means of the same group at different times are significantly different (*p* < 0.05). This figure was drawn using Origin Pro 2021 v. 9.8.0.200.

The initial fluorescence value (Fo) and the maximum fluorescence value (Fm) of mulberry seedlings significantly decreased over time. Figure 2a shows that the Fo values of mulberry seedlings under SS, HS, and FS decreased by 31.27%, 22.51%, and 42.45%, respectively, on day 4 and were significantly different (*p* < 0.05). On days 12 and 16, the Fo of mulberry seedlings under SS increased by 0.85% and 6.87%, respectively, and there was a significant decrease of 9.5% (*p* < 0.05) on the 20th day of waterlogging. The Fo under HS and FS significantly increased, by 12.56% and 25.62%, on the 8th day of waterlogging (*p* < 0.05), respectively, and the Fo under FS showed a significant downward trend at 12–20 days of flooding. Figure 2b shows that Fm under SS, HS, and FS decreased significantly, by 40.54%, 37.67% and 51.6%, respectively, after 4 days of flooding (*p* < 0.05). On day 7, the Fm of mulberry seedling leaves decreased by 12.65% under SS but increased by 28.08% and 40.27% under HS and FS, respectively. The differences under SS, HS and FS were significant (*p* < 0.05). The Fm under SS and HS showed a relatively stable trend in the late stage of flooding, while the Fm under FS showed a significant decreasing trend (*p* < 0.05). In addition, the Fo and Fm of mulberry seedlings under HS were significantly higher than those in SS and FS from days 4 to 16 of flooding.

### Effects of waterlogging stress on the maximum photoperiod quantum yield (QY_max) of PS II and the potential activity of PS II with primary light energy (Fv/Fo) in mulberry leaves

**Figure 3:**
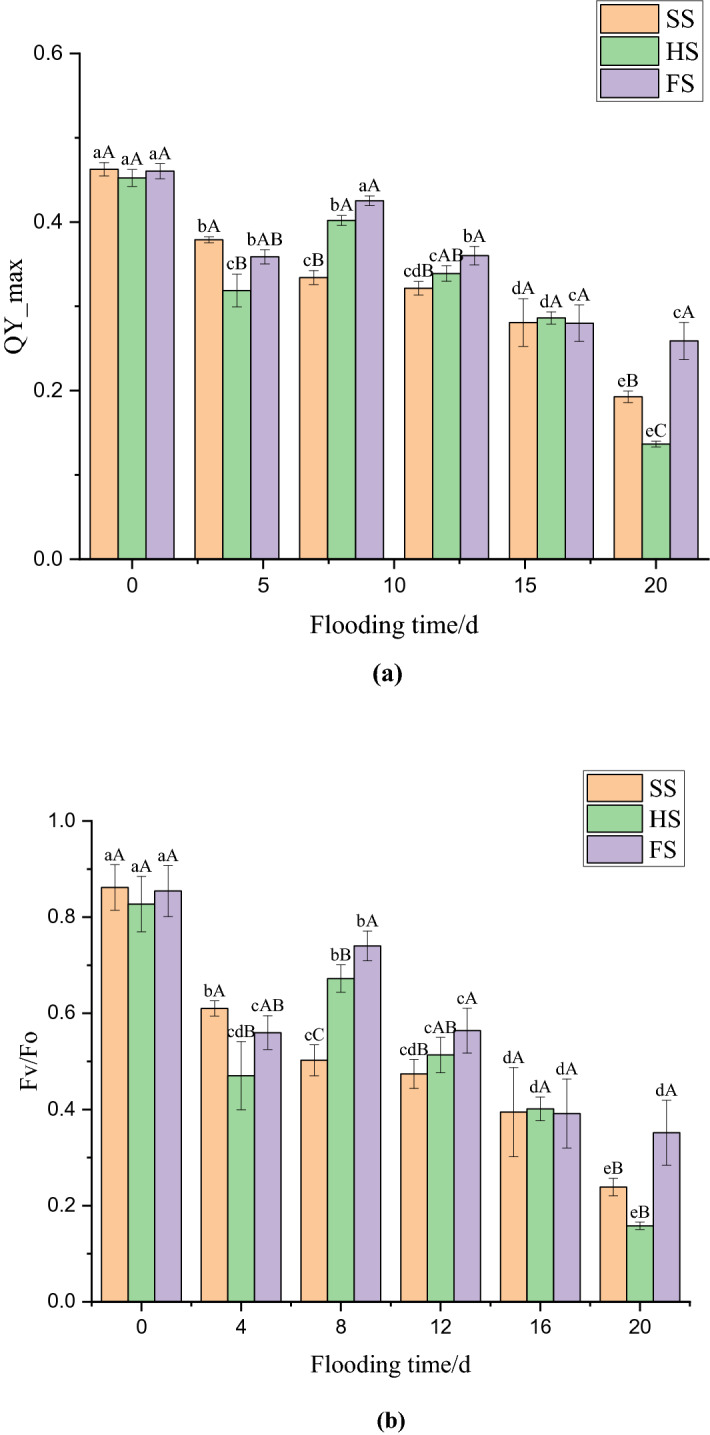
Effect of submergence stress on QY_max and Fv/Fo in *Morus alba.* Different uppercase letters indicate that the means of the different groups at the same time are significantly different (*p* < 0.05), and different lowercase letters indicate that the means of the same group at different times are significantly different (*p* < 0.05). This figure was drawn using Origin Pro 2021 v. 9.8.0.200.

The effects of waterlogging stress at different depths on the maximum photoperiod quantum yield (QY_max) and the potential activity of PS II (Fv/Fo) in mulberry leaves are shown in Fig. 3a,b. After 4 days of waterlogging, the QY_max of mulberry seedlings under SS, HS, and FS significantly decreased (*p* < 0.05), by 18.11%, 29.54%, and 22.1% respectively (Fig. 3a). Under SS, the QY_max of mulberry seedlings continued to decrease during the flooding period, decreasing by 11.82% and 31.39% on the 8th and 16th days, respectively (*p* < 0.05). The QY_max under HS and FS increased significantly, by 26.11% and 18.57%, on day 4 (*p* < 0.05) and decreased by 15.68%, 15.55% and 52.29% under HS on days 12, 16 and 20, respectively (*p* < 0.05). The decreases in QY_max under HS on days 12, 16 and 20 were significant (*p* < 0.05). Under FS, significant decreases in QY_max of 15.31% and 22.25% (*p* < 0.05) occurred on the 12th and 16th days, respectively, and a nonsignificant decrease of 7.54% was observed on the 20th day. Figure 3b shows that the Fv/Fo of mulberry seedlings under SS, HS, and FS decreased significantly, by 29.2%, 43.15% and 34.48%, respectively (*p* < 0.05). On days 8, 12 and 16 during the flooding period, the Fv/Fo of mulberry seedlings under SS decreased by 17.69%, 5.63%, and 16.76%, respectively; it decreased by 39.49% on the 20th day, which was a significant difference (*p* < 0.05). The Fv/Fo of mulberry seedlings under HS and FS significantly increased, by 43.01% and 32.24% respectively, on day 8. However, there was a significant downward trend from days 12–20 of flooding (*p* < 0.05).

### Effects of waterlogging stress on the closure degree (1-qP_Lss) and the rate of steady-state fluorescence decay (Rfd_Lss) of the PS II reaction centre in mulberry leaves

**Figure 4:**
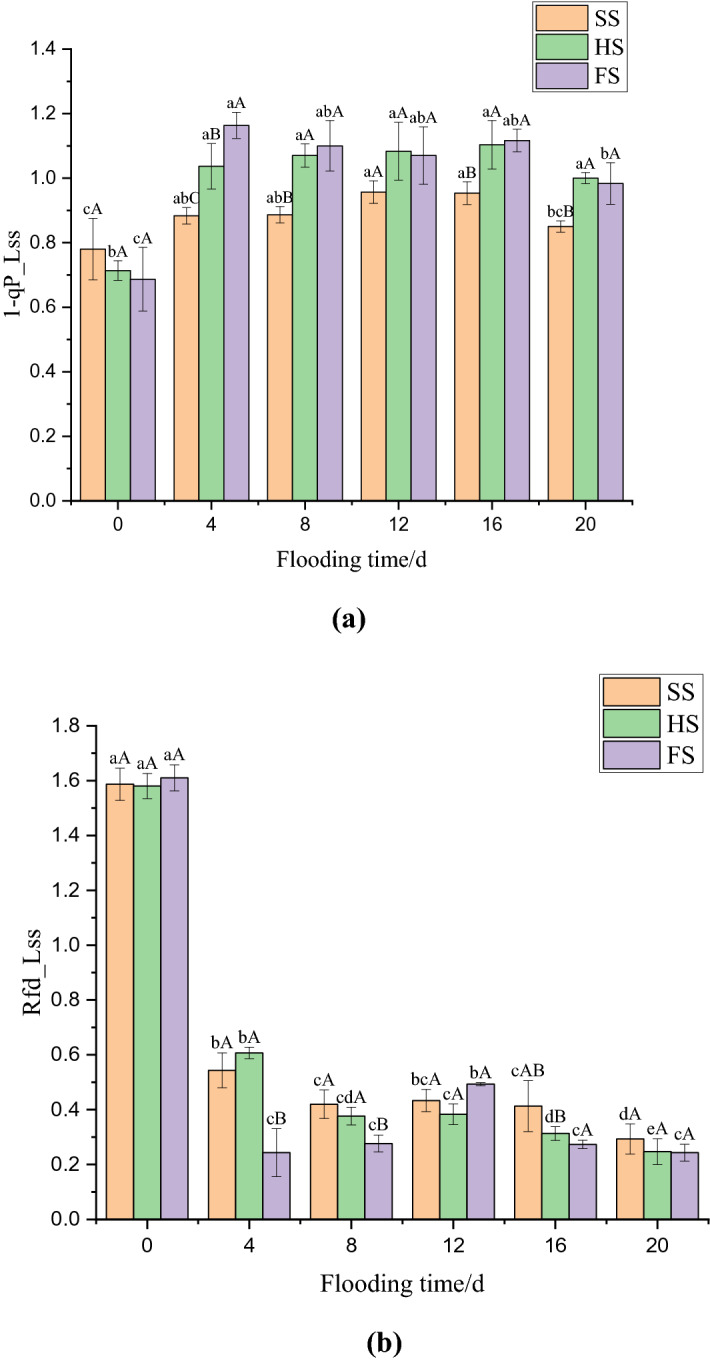
Effect of submergence stress on 1-qP_-_Lss and Rfd_-_Lss values in *Morus alba.* Different uppercase letters indicate that the means of the different groups at the same time are significantly different (*p* < 0.05), and different lowercase letters indicate that the means of the same group at different times are significantly different (*p* < 0.05). This figure was drawn using Origin Pro 2021 v. 9.8.0.200.

The effects of waterlogging stress on the closure degree (1-qP_Lss) and the rate of steady-state fluorescence attenuation (Rfd_Lss) of the PS II reaction centre in mulberry leaves are shown in Fig. 4a,b. The 1-qP_Lss of mulberry seedling leaves increased sharply after 4 days of waterlogging stress, by 13.25%, 45.33%, 69.42% under SS, HS and FS respectively (*p* < 0.05), compared with that before waterlogging (Fig. 4a). The increases in 1-qP_Lss under SS, HS, and FS on day 4 were significant. The 1-qP_Lss in all three groups of 8–16 days of flooding showed a relatively stable trend, and the 1-qP_Lss under SS after 20 days of flooding decreased significantly by 10.84%, compared with that on previous days. In addition, the leaf 1-qP_Lss values under HS and FS were higher than that under SS during the whole flooding period, and there were significant differences between the two groups at 4, 8, 16 and 20 days of flooding (*p* < 0.05). The Rfd_Lss of SS, HS, and FS decreased by 65.76%, 61.6%, and 84.89% respectively after 4 days of flooding (*p* < 0.05), and the values were significantly different (Fig. 4b). The Rfd_Lss values under SS and HS showed a gentle downward trend after 8–20 days of flooding. The Rfd_Lss under FS significantly increased, by 78.31%, after 12 days of flooding and decreased by 45.59% after 16 days of flooding. In addition, the Rfd_Lss values under SS and HS were significantly higher than that under FS (*p* < 0.05) after 4, 8 and 16 days of flooding.

### Effects of waterlogging stress on *Morus alba* chlorophyll fluorescence imaging

**Figure 5:**
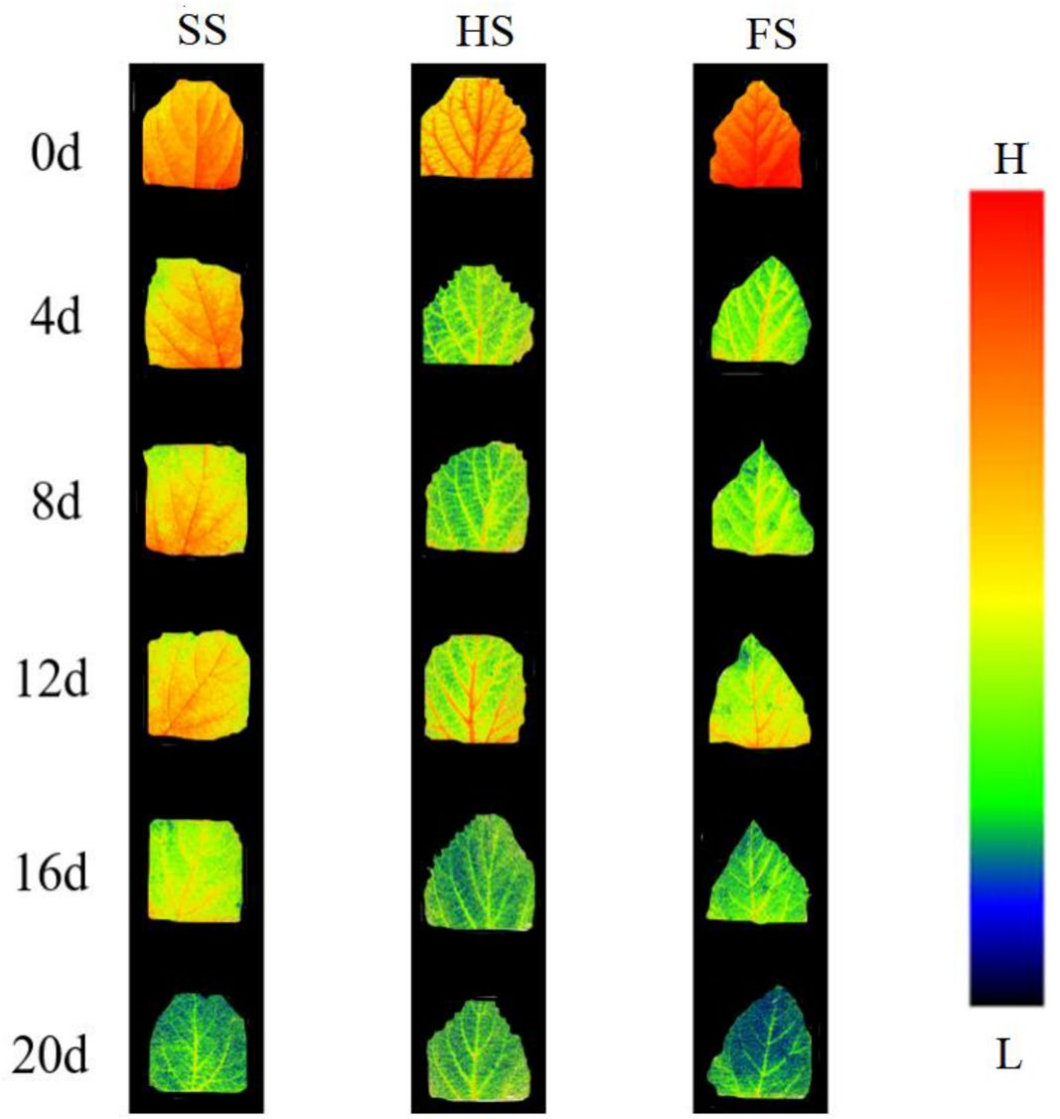
Fliorescence imaging showing the effect of submergence stress on QY_max values in *Morus alba. *The figure was drawn using Photoshop CS6 V13.0.

The fluorescence imaging results for QY_max of mulberry seedlings under different submergence depths are shown in Fig. 5. The results showed that the leaves of mulberry seedlings under the three flooding depth treatments exhibited strong fluorescence values before flooding and that the fluorescence intensity of mulberry seedlings under the flooding depth treatments decreased as the flooding period continued. Among them, the decrease in fluorescence values of mulberry seedlings was the slowest under SS, and there was a significant decrease at 20 days after flooding under SS. The fluorescence values of mulberry seedlings under HS and FS decreased significantly on the 4th day of flooding, and the fluorescence values of FS were significantly lower than those of SS and HS on the 20th day of flooding. In addition, the fluorescence intensity of QY_max at the tip of the mulberry seedling leaves decreased first; then, the fluorescence value at the edge of the blade began to decrease, and the decrease finally extended to the entire leaf blade.

### Correlations between various chlorophyll fluorescence indexes of mulberry leaves under waterlogging stress

Table 1 shows that the various chlorophyll fluorescence indicators were highly correlated. Fm had a very significant positive correlation with Fo, QY_max, Fv/Fo, and Rfd_Lss and a very significant negative correlation with 1-qP_Lss. Fo had a very significant positive correlation with QY_max, Fv/Fo, and Rfd_Lss and a very significant negative correlation with 1-qP_Lss. QY_max has a very significant positive correlation with Fv/Fo and Rfd_Lss and a significant negative correlation with 1-qP_Lss. 1-qP_Lss had a very significant negative correlation with Rfd_Lss.

**Table 1:**
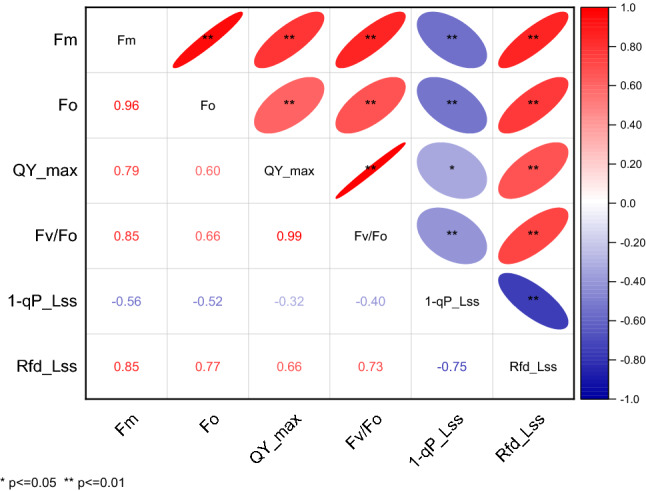
Correlation analysis of the changes in chlorophyll fluorescence indexes of mulberry leaves under different waterlogging treatments. This table was drawn using Origin Pro 2021 v. 9.8.0.200.

## Discussion

The main stress that plants face under flooded conditions is hypoxia^21^. Long-term anaerobic respiration in plants not only reduce the rate of carbohydrate catabolism but also increases the consumption of nutrients in the plant body. The energy production rate of plant tissue under hypoxic conditions was 65–97% lower than that under normal conditions^22^. A study found that plants could reduce the consumption of photosynthetic products by inhibiting leaf growth and reducing the number of blades to adapt to the anoxic environment caused by waterlogging stress^15^. Throughout the flooding process, the mulberry seedlings under SS grew well, and only a small number of leaves started to show leaf curling and slight wilting after 15–20 days of waterlogging. There was no change in the morphological appearance of the mulberry seedlings under HS after 5 days of waterlogging, and slight wilting and shedding occurred after 10 days of waterlogging. Yellow–brown spots appeared on the leaves of mulberry seedlings under FS after 10 days of waterlogging, and the wilting, yellowing and shedding of the leaves became increasingly serious with increasing waterlogging time. The results showed that the effects of the three different depths of waterlogging stress on the morphology of mulberry seedling leaves were as follows: FS > HS > SS. This is basically consistent with the result that 1-year-old mulberry leaves showed mild symptoms after long-term root waterlogging stress but showed obvious symptoms under severe waterlogging stress on the 5th day of submergence^23^. In addition, it has been found that there are two opposite adaptive strategies by which plants adapt to waterlogging stress: the quiescence strategy and the deep-water escape strategy^21^. In the quiescence strategy, plants reduce their nutrient consumption by inhibiting the growth of their aerial parts to prolong their survival time^24^. In the deep-water escape strategy, plants facilitate the internal O_2_ diffusion by forming aerenchyma so that O_2_ can diffuse to the O_2_-deprived plant parts. At the same time, plants can outgrow the water through increased shoot elongation to obtain sufficient O_2_^25^. The formation of adventitious roots from plant stems can improve the transportation of nutrients and oxygen^26^, supplement or even replace the primary roots that die from a lack of oxygen^11^, and enhance the flooding tolerance of plants. A study found that waterlogging stress could result in the formation of adventitious roots in plants such as *Oenanthe javanica* (Bl.) DC., *Polygonum hydropiper* Linn., *Amorpha fruticosa* Linn., *Distylium chinense* (Fr.) Diels, and *Cyperus rotundus* Linn.^15,27–30^. The results of this study showed that mulberry seedlings under HS produced adventitious roots on the 10th day after flooding and that the number of adventitious roots increased with increasing flooding time. However, the mulberry seedlings under HS produced only a small number of adventitious roots on the 15th day after flooding. This may have occurred because the adventitious roots formed from the stems of mulberry seedlings under HS were exposed to the water surface, with has a higher oxygen content than other water layers and thus alleviated hypoxia stress. However, it was difficult for the adventitious roots of mulberry seedlings under FS to absorb oxygen from water with low dissolved oxygen content^31^. This is similar to previous results showing that the number of adventitious roots of *Salix variegata* Franch. under root-flooding conditions increased with increasing flooding time, while the number of adventitious roots under full flooding conditions was very low^31^.

Changes in chlorophyll fluorescence parameters can be used to characterize the adaptability and tolerance of plants to changes in the growth environment^32^. Studies have found that the photosynthetic reaction centre of plants under nonstress conditions are more functional than that of plants under stress conditions. Therefore, plants grown under nonstress conditions have higher fluorescence parameters such as Fv/Fm and qP^33^. The Fo is an important indicator to evaluate the stress injury of plants, and Fm after dark adaptation can reflect the PS II electron transport capacity^34,35^. The change in Fv/Fo indicates that the light energy absorbed by PS II was used to reduce the efficiency of QA and the potential vitality changes in PS II^36^, which can reflect the tolerance of plants to adverse environments^34^. The Rfd-Lss can be used to assess the vigour of plants. The results of this study showed that the Fo, Fm, Fv/Fo and Rfd-Lss of mulberry seedlings treated with different water depths were significantly reduced at the early stage of waterlogging stress, which indicating that the early stage of waterlogging stress will lead to a decrease in the activity of the PS II reaction centre in mulberry seedling leaves. The Fo, Fm, and Fv/Fo of mulberry seedlings under HS and FS increased significantly after 8 days of flooding, which indicated that both semiflooded and deep-flooded conditions caused the reversible inactivation of the PS II reaction centre of the leaf, resulting in photoinhibition. The photosynthetic system of leaves can self-repair by increasing the electron transfer efficiency and heat dissipation of PS II, to reduce the damage to the photosynthetic mechanism caused by waterlogging stress. It can be seen from the results that the increase in fluorescence parameters under FS was more significant than that under HS. However, the Fo, Fm and Fv/Fo of the mulberry seedlings under SS showed a relatively stable downward trend in the late flooding period, which indicated that an increase in flooding depth and a prolongation of the flooding time would aggravate the damage to photoreaction centre activity in mulberry seedling leaves.

The 1-qP_Lss can reflect the redox state of the PS II original electronic receptor QA to some extent^37^ and can be used as an indicator to determine the occurrence of photoinhibition^34^. The results of this study showed that the 1-qP_Lss of mulberry seedling leaves in the three groups increased significantly in the early stage of flooding. The degree of elevation was as follows: FS > HS > SS. This indicated that waterlogging stress damaged the PS II reaction centre of mulberry seedlings, reducing its ability to accept electrons, and that the degree of damage was proportional to the submergence depth. The degree of PS II reaction centre closure in mulberry seedling leaves decreased after 20 days of flooding, which may be the result of the self-healing of the photosynthetic system after adaptation to the flooded environment. In addition, the QY_max can reflect the efficiency and utilization of excitation energy captured by open PS II reaction centres, which is an important indicator that reflects the degree of damage to the plant PS II reaction centre^35^. Therefore, QY_max fluorescence imaging effectively reflected the damage to the PS- II reaction centre of mulberry seedlings under waterlogging stress. The imaging results showed that the QY_max of mulberry seedlings decreased significantly under waterlogging stress, which may be related to the redistribution of the fraction of light energy used for photochemical electron transport due to the increase in 1-qP-Lss^37^. In addition, with increasing flooding time, the fluorescence intensity of mulberry seedling leaves decreased continuously, and the decreasing trend was as follows: FS > HS > SS. This indicated that increases in both flooding time and depth would aggravate the damage to the PS II reaction centre of mulberry seedlings. This result is similar to the finding that the QY_max of tomato plants (*Lycopersicon esculentum*) decreased significantly after they were exposed to high-concentration, short-term alkali stress or low-concentration, long-term alkali stress^38^. Fluorescence imaging showed that the decrease in fluorescence intensity in mulberry leaves under waterlogging stress began at the tip of the leaves, and then spread gradually from the edge of the leaves to the entire leaf. In addition, after the end of the waterlogging stress period, strong fluorescence was still observed in the veins of the mulberry leaves under the different treatments, which may be related to the vascular bundle structure of the veins. The vein structure is different from the leaf structure, and it is speculated that the vascular bundle structure may be more difficult to destroy than the leaf structure^35^.

## Conclusions

After 20 days of waterlogging stress, the leaves of the mulberry seedlings under SS were healthy, and the mulberry seedlings in the HS and FS groups showed different degrees of flooding stress characteristics at the late stage of flooding. The mulberry seedlings in the FS and HS groups produced adventitious roots after 10 and 15 days of flooding, respectively, and the number of adventitious roots in the HS group was significantly higher than that in the FS group. Fo, Fm, QY_max, Fv/Fo, and Rfd_Lss decreased significantly and 1-qP_Lss increased significantly in the early stage of waterlogging stress, suggesting that waterlogging stress can damage the photosynthetic apparatus of mulberry leaves, hinder electron transfer and reduce the activity of the PS II reaction centre. Furthermore, the results indicated that increases in the time and depth of the flooding test conditions aggravated the damage to the mulberry leaves. In the later stage of flooding, the fluorescence parameters showed a relatively stable trend which was the result of the self-repair of the photosynthetic system in mulberry seedling leaves as an adaptation to the flooding environment. In addition, the changes in the leaf morphology and chlorophyll fluorescence characteristics of mulberry seedlings under short-term waterlogging stress were studied in the nongrowth season. It is necessary to carry out research on the changes in the fluorescence characteristics of mulberry seedlings during the growth season and under long-term waterlogging stress.

## Materials and methods

### Growth conditions

The test materials were obtained from the Canghai mulberry seedling base in Dalangba, Qukou town, Kai County, Chongqing, China, and were Shengsang No.1 3-year-old forage mulberry seedlings grown naturally in a mulberry forest. On June 1, 2017, 100 Shengsang No.1 forage mulberry seedlings (average plant height, 22.26 cm; average ground diameter, 15.69 mm) were planted into pots with an inner diameter of 30 cm and a height of 23 cm (one plant per pot) and placed in Beijing Bajia Sanqingyuan Nursery (40° 01′ N, 116° 33′ E). The nursery is located in the northwestern part of the North China Plain, which has a temperate monsoon climate with an annual average temperature of 12.5 °C and a multiyear average precipitation of 628.9 mm. The cultivated soil was a yellow–brown soil brought back from the Chongqing mulberry seedling base with a compactness of 1.96 MPa; a total porosity of 36.2%; a bulk weight of 1.584 g/cm^3^; total potassium, phosphorus, and nitrogen mass fractions of 1.63%, 0.071%, and 0.104%, respectively; and a soil pH of 5.5–6.5. To ensure the normal growth of the seedlings, typical watering and management practices were carried out.

### Experimental design

On 1 October 2017, mulberry seedlings with good, uniform growth were selected to carry out the waterlogging stress tests. The average height of the mulberry seedlings was 37.33 cm and the average diameter of the mulberry seedlings was 17.14 mm. The mulberries were divided into groups and subjected to three flooding intensities: a shallow submergence treatment (SS), with the flooding depth exceeding the soil height in the pot by 2 cm; a half-submergence treatment (HS), with the flooding depth exceeding the soil height in the pot by 19 cm; and a full-submergence treatment (FS), with the flooding depth exceeding the soil height in the pot by 52 cm. Five mulberry seedlings were grown in each treatment group, and the flooding conditions were controlled by plastic water tanks of the same size (tank size: 98 cm in length, 76 cm in width, and 68 cm in height). To simulate the constantly flowing water in the TGRA, the water in the tank was changed every 5 days after the beginning of flooding. The waterlogging stress ended on 21 October 2017 and lasted for 20 days.

### Observation of external morphological changes

The morphological changes in the mulberry leaves (including the number of curled or withered leaves, the number of yellow leaves, the number of defoliated leaves, the number of brown spots or rotten leaves, etc.) and the formation of adventitious roots were observed every 5 days after the onset of waterlogging stress, and photographs of the seedlings were taken. Five mulberry seedlings were placed in each treatment group for morphological observation.

### Determination of the kinetic parameters of chlorophyll fluorescence

Table 2 shows the symbols and definitions of chlorophyll fluorescence indexes. The fluorescence parameters and images were determined at 7:00–11:00 a.m. after 0, 4, 8, 12, 16 and 20 days of submergence. The chlorophyll fluorescence measurements were taken using a Fluorcam portable chlorophyll fluorescence imager (Handy Fluorcam FC 1000-H, PSI, Brno, Czech Republic) from the top 3–5 fully expanded leaves of the mulberry seedlings, which revealed the PS II activity^39,40^. The parameters are set to shutter, 1; sensitivity, 28%; super, 90%; far, 50%; act, 1 100%; and QY_max, a range of 0–0.67. The fluorescence parameters measured were the initial fluorescence (Fo), maximum fluorescence (Fm) after dark adaptation, maximum light quantum efficiency (QY_max), closure degree of the PS II reaction centre (1-qP_Lss) under a steady-state, the steady-state fluorescence decay rate (Rfd_Lss), and the corresponding chlorophyll fluorescence images. Three replicate measurements were taken for each treatment as follows. Prior to the measurements, the mulberry seedlings were put into jars covered with foil for 20 min of dark adaptation. The minimal Chl fluorescence Fo was determined with low-intensity measuring light pulses (620 nm). Then, a 0.8 s saturating pulse of white light (3000 μmol photons m^−2^ s^−1^) was applied to determine the maximum fluorescence in a dark-adapted state, Fm. After 27 s of dark adaptation, the leaves were exposed to actinic white light (200 μmol photons m^−2^ s^−1^) for 60 s until Ft_Lss was reached. The value of Fp was recorded when the actinic light was irradiated, followed by 60 s of dark relaxation. Fm_Lss, the steady-state maximum fluorescence in light, was determined using a series of 0.8 s pulses of saturating white light. Fo_Lss, the steady-state minimum fluorescence in light, was determined with low-intensity light pulses (620 nm).

**Table 2:** Detailed information on the chlorophyll fluorescence indexes.

Symbol	Formula	Name	Description
Fo	Measured	Minimum fluorescence in a dark-adapted state	QA oxidized (qP = 1), non-photochemical quenching relaxed (NPQ = 0)
Fm	Measured	Maximum fluorescence in a dark-adapted state	QA reduced (qP = 0), non-photochemical quenching relaxed (NPQ = 0)
QY_max	Fv/Fm	Maximum quantum yield of PS II	Maximum PS II quantum yield in a dark-adapted state
Fv/Fo	Fm-Fo/Fm	Potential photosynthetic activity of PS II	The change in PS II-absorbed light energy to reduce the efficiency of QA and potential PS II activation
1-qP_Lss	1 − [(Fm_Lss − Ft_Lss)/(Fm_Lss − Fo_Lss)]	Closure degree of the PS II reaction centre under a steady state	Estimate of the fraction of closed PS II reaction centres PS II closed/(PS II open + PSII closed)
Rfd_-_Lss	(Fp − Ft_Lss)/Ft_Lss	Steady-state fluorescence decline ratio	Empiric parameter used to assess plant vitality

### Analytical methods

The obtained experimental data were compiled with Microsoft Excel 2017, plotted with Origin Pro 2021 software, and analysed through ANOVA and correlation analysis with SPSS 22.0 software. Multiple comparisons were performed with Duncan’s method.

### Ethics declarations

We had obtained permission to collect plants. All experiments were performed in accordance with relevant guidelines/regulations.
